# A Multidomain Lifestyle Intervention Is Associated With Improved Functional Trajectories and Favorable Changes in Epigenetic Aging Markers in Frail Older Adults: A Randomized Controlled Trial

**DOI:** 10.1111/acel.70376

**Published:** 2026-02-12

**Authors:** Gloria Olaso‐Gonzalez, Fernando Millan‐Domingo, Luis Garcia‐Fernandez, Elisa Garcia‐Tercero, Monica Cebrian, Cristina Garcia‐Dominguez, Juan Antonio Carbonell, German Casabo‐Valles, Jose Luis Garcia‐Gimenez, Eva Tamayo‐Torres, Juan Gambini, Francisco Jose Tarazona‐Santabalbina, Jose Vina, Maria Carmen Gomez‐Cabrera

**Affiliations:** ^1^ Department of Physiology, Faculty of Medicine University of Valencia Valencia Spain; ^2^ INCLIVA Health Research Institute Valencia Spain; ^3^ CIBER de Fragilidad y Envejecimiento Saludable (CIBERFES) ISCIII Madrid Spain; ^4^ Body, Physical Activity and Sport Study Group (GECAFD), Sports Department, Universidad Industrial de Santander Bucaramanga Colombia; ^5^ Department of Geriatric Medicine Hospital Universitario de la Ribera Valencia Spain; ^6^ Instituto de Investigación Sanitaria La Fe Valencia Spain; ^7^ Department of Internal Medicine Hospital Royo de Villanueva Zaragoza Spain; ^8^ EpiDisease S.L. Scientific Park, University of Valencia Paterna Spain; ^9^ CIBER de Enfermedades Raras (CIBERER) ISCIII, Madrid Madrid Spain; ^10^ Medical School Universidad Católica de Valencia San Vicente Mártir Valencia Spain

**Keywords:** epigenetic clock, exercise, frailty, healthy aging, nutrition, telomere length

## Abstract

Frailty emerges as the intermediate stage preceding disability, but there is a gap in molecular signatures for early detection of subclinical cellular changes, which could help predict frailty onset or the effectiveness of interventions. In this randomized, controlled study, we assessed phenotypical and functional changes in frail individuals before and after a 6‐month multidomain lifestyle intervention (nutritional supplement and supervised exercise) vs habitual care. We also analyzed whole‐blood methylome, including five epigenetic clocks, a DNA methylation‐based telomere length estimator, and the Rate of Epigenetic Aging (REA). Between October 2019 and July 2022, we recruited 47 frail, community‐dwelling individuals in Spain. Mean age was 80.2 years (SD 3.1) in the control group (CG; *n* = 19) and 80.5 years (4.3) in the intervention group (IG; *n* = 28). Compared with the CG, a significant reduction in frailty, assessed by the SHARE‐FI score, was observed in the IG (*p* < 0.0001). The IG also showed improved grip strength (*p* = 0.0053), gait speed (*p* = 0.0125), the Tinetti score (*p* = 0.0031), and Barthel Index (*p* = 0.0484). The intervention was also associated with statistically significant improvements in nutritional blood markers, indicators of biological aging, including reduced DNAm PhenoAge (*p* = 0.0253) and preserved telomere length (*p* = 0.0246). REA using DNAm PhenoAge indicated an acceleration of epigenetic aging in the CG (*p* = 0.0300). Other epigenetic clocks showed nonsignificant changes. Our findings suggest potential geroprotective effects of a multidomain intervention and indicate that DNAm PhenoAge and methylation‐based telomere length may serve as complementary markers for assessing health span‐related changes in frail older adults.

**Trial Registration:** This trial was retrospectively registered: NCT06975540

## Graphical abstract

In frail older adults, a 6‐month lifestyle intervention slowed epigenetic aging and improved functional outcomes, suggesting lifestyle strategies may delay biological aging and disability progression.

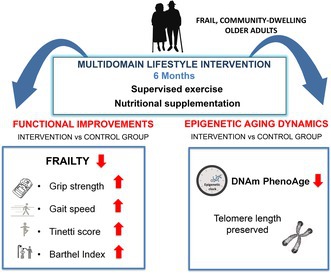

## 1 Introduction

Frailty is a state of diminished capacity to handle stressors resulting from a decline in functional reserves (Fried et al. 2004; Arc‐Chagnaud et al. 2019). Frailty exhibits a higher predictive value than chronic diseases for adverse outcomes among older individuals. An essential determinant of frailty is having an unhealthy lifestyle, characterized by poor dietary habits, low levels of physical activity, and obesity (Strandberg et al. 2012), which together accelerate physiological decline (Rodriguez‐Mañas and Fried 2015). These modifiable factors offer critical opportunities for prevention and intervention to delay or mitigate the onset of frailty (Millan‐Domingo et al. 2022). However, while assessment of modifiable risk factors is important, such measures capture downstream or overt manifestations of vulnerability and may not fully reflect early, subclinical biological dysregulation.

A critical gap in frailty research is the lack of validated molecular signatures capable of identifying individuals at risk before functional impairment becomes clinically evident or of predicting heterogeneity in response to interventions (Cummings and Kritchevsky 2022). Molecular biomarkers may provide complementary information on underlying cellular and molecular processes, improving early detection, risk stratification, and the evaluation of intervention effectiveness beyond what can be achieved through lifestyle and clinical assessments alone.

Epigenetic clocks are DNA methylation‐based algorithms that analyze genome‐wide methylation patterns to estimate biological aging relative to chronological age (Horvath 2013). Methylated CpG islands in gene promoters have been associated with long‐term gene silencing and are related to transcriptomic changes (García‐Giménez et al. 2021). Because the methylome operates at the interface between the genome and the environment, it can shift in response to environmental stimuli, such as pollutants (Seale et al. 2022), exercise, or diet (Barrès et al. 2012; Urdinguio et al. 2021).

The first‐generation epigenetic clocks (Horvath's and Hannum's clocks) were originally trained to determine chronological age (Hannum et al. 2013; Horvath 2013). More recent epigenetic clocks were developed with the aim of predicting biological age, particularly morbidity and mortality risk (second‐generation clocks as DNAm PhenoAge and DNAm GrimAge) or even to predict functional capacity, rate of aging, and health span trajectories (third‐generation clocks as DNAm FitAge) (Levine et al. 2018; Lu et al. 2022; McGreevy et al. 2023).

Few longitudinal and lifestyle‐based interventional studies are available for epigenetic clocks. These clocks have been utilized to assess the impact of various drug interventions in humans (Fahy et al. 2019) and of caloric restriction (Waziry et al. 2023).

Our prospective study aimed to evaluate a multidomain lifestyle intervention vs habitual care to reverse frailty among community‐dwelling older adults. Our intervention included a daily nutritional supplement intake and a supervised and personalized multicomponent exercise program 3 days a week for 6 months. Apart from a deep phenotypical and functional characterization with pre‐ and post‐intervention assessments, we performed a parallel methylome analysis to characterize epigenetic changes in whole blood cells. This included five epigenetic clocks (Horvath, Hannum, PhenoAge, GrimAge, and FitAge), a DNAm‐based telomere length estimator, and the REA.

This study integrates well‐established biomarkers of biological aging with comprehensive assessments of phenotypic and functional domains in a longitudinal design in old, frail individuals.

## 2 Methods

Our study is a prospective, interventional, controlled, randomized clinical study (protocol number MEJ‐NES‐2019). The researchers responsible for the exercise intervention and data gathering were independent to guarantee the study's blindness. Assessors were not informed of participants' assignment. The study was conducted between October 14, 2019, and July 29, 2022. Informed consent was obtained from each participant who signed it after fully understanding the procedures (*n* = 47).

Eligibility criteria included: (i) age ≥ 70 years; (ii) sedentary lifestyle per WHO (WHO 2020); (iii) frail according to the Survey of Health, Aging and Retirement in Europe‐Frailty Instrument (SHARE‐FI) (Romero‐Ortuno et al. 2011); (iv) gait speed ≤ 0.8 m/s; and (v) community dwellers.

**FIGURE 1:**
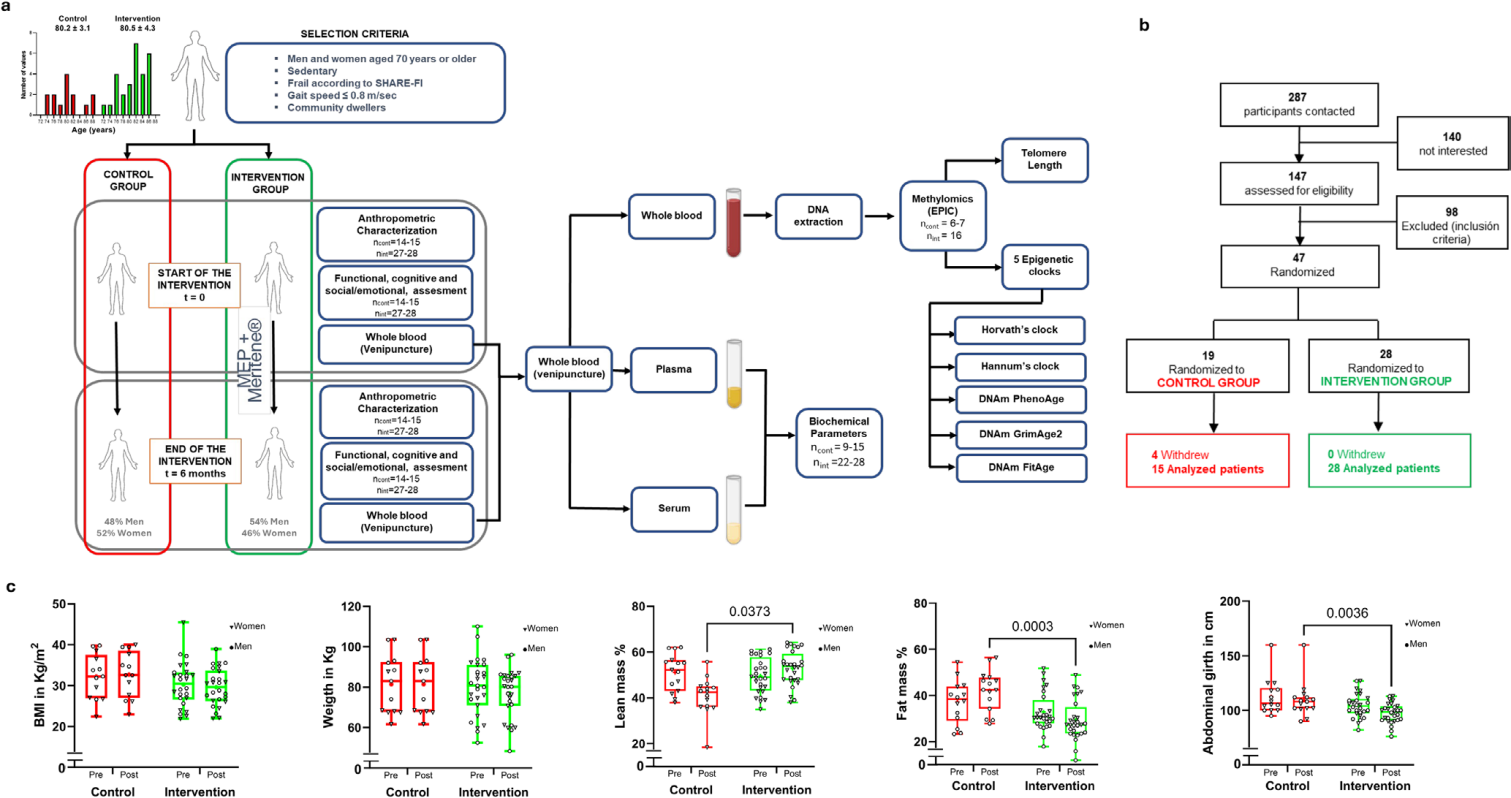
Study design and anthropometric characteristics of the participants. (a) Overview of selection criteria and study design. The histogram represents the age distribution in the control group (*n* = 19) and the intervention group (*n* = 28). (b) Flowchart of participant selection and progression. (c) Main anthropometric data of participants before and after the intervention, including BMI, percentage of lean mass, percentage of fat mass, and abdominal girth (*n*_cont_ = 14–15, *n*_int_ = 27–28). Inverted triangles represent female participants, while circles represent male participants. A two‐sided ANOVA test was used to assess statistically significant differences.

Exclusion criteria were: (i) life expectancy < 12 months; (ii) uncontrolled diabetes (HbA1c > 9%); (iii) cognitive impairment (MMSE < 17); (iv) disability (Barthel Index < 50); (v) recent acute coronary event; (vi) hospital admission in past 3 months; (vii) active cancer treatment; (viii) major surgery in past 6 months; (ix) institutionalization; (x) NYHA class 3–4 dyspnea; (xi) lactose intolerance; (xii) use of multivitamins or protein supplements; (xiii) refusal to consent. Baseline sample characteristics are shown in Figure 1 and Table S1.

### 2.1 Randomization and Power Calculation

The listing of individuals was obtained from the Valencian Community health database and participants were recruited primarily through telephone contact. Forty‐seven community‐dwelling frail older adults (age ≥ 70) were recruited and divided into two experimental groups: a control group (*n* = 19) and a multidomain (exercise training and nutritionally supplemented) group (*n* = 28). The towns where the intervention and control programs were conducted were randomly assigned using simple randomization procedures performed by the Department of Research of the Health Department in La Ribera. Participants were randomly assigned to the intervention or control group using a 3:2 allocation ratio (intervention:control). Participants assigned to the control group received habitual care and did not receive specific nutritional or exercise advice, nor any additional contact beyond the standard follow‐up assessments. Figure 1 outlines the subjects flow diagram from first contact to study completion.

With the final sample size achieved at the end of the study, 15 participants in the control group and 28 in the intervention group, the statistical power for detecting differences in the SHARE‐FI score was 1.0.

The effect size in SHARE‐FI was 3.2, calculated from group means of 4.0 and 0.8, assuming a standard deviation of 1.0 in each group. The comparison was performed using a two‐tailed Wilcoxon–Mann–Whitney test and G*Power version 3.1.9.7.

### 2.2 Multidomain Intervention

The intervention group followed a multidomain intervention consisting of a specific nutritional supplement and a multicomponent exercise program.

#### 2.2.1 Nutritional Intervention

It included a daily intake of two servings of Meritene Strength and Vitality nutritional supplement (ready‐to‐mix powder ONS). Each serving (35 g of powder diluted in 200 mL of partially skimmed milk) provides 199 kcal, 16 g of high‐quality protein, and 19 essential vitamins and minerals (Nestlé Health Science, Switzerland).

#### 2.2.2 Multicomponent Exercise Program

The multicomponent exercise program consisted of supervised, group‐based sessions conducted three times per week for 24 weeks, each lasting 60 min. The intervention included 5 min of warm‐up, 20 min of progressive resistance training (ranging from 45%–55% to 70%–75% of one‐repetition maximum), 20 min of cardiorespiratory exercise (55%–75% of maximum heart rate), 10 min of neuromotor and balance exercises, and 5 min of stretching. All sessions were supervised by a sport scientist (Tarazona‐Santabalbina et al. 2016; Millan‐Domingo et al. 2022).

The exercise sessions targeted the major muscle groups of both the upper and lower limbs, as well as core stability and postural control. Resistance training was performed using elastic bands and bodyweight exercises, including concentric, eccentric, and isometric contractions that engaged the quadriceps, hamstrings, gluteal muscles, biceps, triceps, and forearm flexors and extensors. Cardiorespiratory training consisted of walking circuits and stair climbing. Neuromotor exercises emphasized balance, proprioception, and coordination through dynamic postural control and flexibility work of the lumbopelvic region. Stretching exercises focused on the neck, upper and lower limbs to promote and maintain joint mobility.

The training program was conducted during the COVID‐19 pandemic, with strict adherence to hand hygiene, mask‐wearing, and social distancing measures by both patients and trainers.

### 2.3 Safety and Tolerability

Safety and tolerability were monitored throughout the course of the study for all groups.

### 2.4 Measurements

The primary outcome in our trial was the change in frailty status after 180 days, assessed using the SHARE‐FI (Romero‐Ortuno et al. 2010). We also included the evaluation of the Fried's frailty criteria (Fried et al. 2001). Both instruments evaluate five core domains of the frailty phenotype: fatigue, unintentional weight loss or reduced appetite, weakness (grip strength), slowness, and low physical activity. These domains are measured through a combination of self‐reported items and an objective assessment of grip strength, providing both a continuous frailty score and a categorical classification (non‐frail, pre‐frail, or frail) based on sex‐specific cut‐off values.

Collected data included age, gender, marital status, and anthropometric measurements (BMI, abdominal, brachial, and calf girths). Body composition was measured via bioelectrical impedance (Tanita BC‐601). Nutritional status was evaluated with the MNA‐LF (Guigoz and Vellas 2021). Functional status included Barthel, Lawton and Brody, Tinetti, and handgrip strength. Cognitive, emotional, and social aspects were assessed using the MMSE, Duke, EQ‐5D, and Yesavage scales; age‐related conditions, geriatric syndromes, and healthcare usage were also recorded.

### 2.5 Biochemical Analysis

Serum and plasma were collected before and after the intervention and stored at −80°C. A complete hematological and biochemical analysis was performed in the hospital's clinical laboratory following standard methods (See Table S3).

### 2.6 DNA Extraction

Genomic DNA was extracted from whole blood using Chemagic DNA Blood 400 Kit H96 (PerkinElmer, Waltham, MA, USA) and the Chemagic 360 Instrument (PerkinElmer, Waltham, MA, USA) following the manufacturer's instructions.

### 2.7 DNA Bisulfite Conversion

The DNA bisulfite conversion was performed using the EZ DNA Methylation Kit (Zymo Research, Cat. No.: D5001; Irvine, USA). For each sample, 500 ng of DNA was used and normalized to a concentration of 12 ng/μL by adding the required amount of H_2_O to achieve a final volume of 45 μL.

### 2.8 DNA Methylation Analysis

The DNA methylation analysis was performed using the Infinium MethylationEPIC v2.0 BeadChips (Illumina Inc., San Diego, CA, USA), which target over 930 k unique methylation sites in the most biologically significant regions of the human methylome. Samples were hybridized at 48°C for 16 h. Unbound and non‐specific DNA was washed away. A single‐base extension was performed using labeled nucleotides (biotin and DNP), followed by fluorescent staining with specific antibodies. The BeadChip was then washed, protected, and scanned on the Illumina HiScan SQ.

### 2.9 Bioinformatic Analysis

The IDAT files from Illumina arrays were processed using the *minfi* R package (Aryee et al. 2014). Quality control removed samples with poor hybridization (mean detection *p*‐value > 0.05) and redundant paired samples. Cell‐type proportions were estimated using the FlowSorted.Blood.EPIC package (Salas et al. 2018). Data were normalized using quantile normalization. Probes with low detection, on sex chromosomes, with SNPs, or with cross‐reactivity were excluded.

### 2.10 Analysis of DNAm Clocks and Pace of Aging Measures

Epigenetic age was estimated using the *methylclock* R package (Pelegí‐Sisó et al. 2021) for Horvath's (Horvath 2013), Hannum's (Hannum et al. 2013) and DNAm PhenoAge (Levine et al. 2018b) clocks. A total of 31, 12, and 13 CpG sites were missing for these clocks, respectively, and were excluded from the estimations. DNAm GrimAge2 (Lu et al. 2022) and DNAm FitAge (McGreevy et al. 2023) are not currently available for calculation in this package and were computed using the online DNAm Age Calculator (Horvath 2013). All 1331 GrimAge2 probes were used for the estimation, while 3 of 789 FitAge probes were missing and therefore not considered. All epigenetic clocks were computed following their original published algorithms and preprocessing procedures. Although data originated from different DNA methylation array platforms, cross‐platform validation studies indicate that this has minimal impact on clock comparability (Föhr et al. 2021; Apsley et al. 2025; Tay et al. 2025).

### 2.11 Rate of Epigenetic Aging

To assess the longitudinal dynamics of epigenetic aging, we calculated the REA, defined as the ratio of the difference between epigenetic age at the follow‐up time point (6 months) and the beginning of the study (baseline) to the difference between chronological age for the same time points, as previously reported (Sehl et al. 2021; Schoepf et al. 2023).

$$\begin{array}{c} \text{Rate of Epigenetic aging}=\left[\text{Epigenetic}\;\mathrm{age}\;\left(6\;\text{months}\right)\right]\\ –\left[\text{Epigenetic}\;\mathrm{age}\;\left(\text{Baseline}\right)\right]/\left[\text{Chronological}\;\mathrm{age}\;\left(6\;\text{months}\right)\right]\\ –\left[\text{Chronological}\;\mathrm{age}\;\left(\text{Baseline}\right)\right] \end{array}$$

### 2.12 Epigenetic Age Acceleration

Age Acceleration was calculated by regressing DNAm PhenoAge on chronological age across all participants (Horvath et al. 2015). The residuals from this model, representing the difference between observed and predicted DNAm age, were used as Age Acceleration values. Positive values indicate accelerated epigenetic aging relative to chronological age.

### 2.13 Statistical Analysis

Statistical analysis was performed using GraphPad Prism (Version 10). The normality of the data was assessed using the Shapiro–Wilk or Kolmogorov–Smirnov tests. For normally distributed variables comparisons were performed using a paired *t*‐test, while between‐group differences were analyzed with an independent samples *t*‐test. For variables that were not normally distributed, the Wilcoxon signed‐rank test was used for paired (related) samples. The Mann–Whitney U test was applied to compare independent groups. A two‐way ANOVA was conducted to analyze the interaction between two independent variables (time and intervention). Correlation between age and epigenetic age was carried out using Spearman's rho statistic. Factor Analysis of Mixed Data (FAMD) was performed using R version 4.4.1 and the *PCAmixdata* package.

Data were examined for outliers, which were excluded based on predefined statistical criteria before analysis. Analyses were conducted on an available‐case basis, including all participants with valid data for each outcome. Sample sizes varied accordingly, and missing data were not imputed. Except for methylomic outcomes, obtained in a smaller subsample, missingness was low.

Statistical significance was set at a *p*‐value < 0.05. No adjustment for multiplicity was carried out. Frailty score, assessed using the SHARE‐FI, was designated as the primary outcome of the study. All other measured variables were considered secondary or exploratory outcomes.

## 3 Results

### 3.1 Study Participants

Figure 1a shows an overview of selection criteria and study design. The histogram represents the distribution of ages among the control group (*n* = 19) and intervention group (*n* = 28).

We screened 287 potential participants telephonically; 147 were assessed for eligibility, 47 were recruited and randomized, 28 to the intervention, and 19 to the control group (Figure 1b). Baseline characteristics of the participants are available in Table S1. We found no relevant differences between the groups in the main conditions studied or in the prevalence of geriatric syndromes. The intervention group did not differ from controls in terms of gender (*p* = 0.4), weight (*p* = 0.9), BMI (*p* = 0.3) or chronological age (*p* = 0.8) (Figure 1a,c). No significant differences were found in either the SHARE‐FI index or Linda Fried's frailty criteria (Oviedo‐Briones et al. 2021).

No adverse events or health complications related to the exercise program or nutritional supplementation were observed during the study period.

### 3.2 Effect of a Six‐Month Multidomain Intervention on Functional, Cognitive, Emotional, and Social Losses in Frail Individuals

Figure 1c and Table S2 show that the anthropometric baseline characteristics of the two groups were very similar at the beginning of the study. Our multidomain intervention significantly improved lean mass, body fat percentage, and reduced abdominal girth. Participants' exercise compliance was 64.3% (95% CI 49.8–68.6). Adherence to the nutritional supplement was 71.2% (95% CI 67.5–74.6).

**FIGURE 2:**
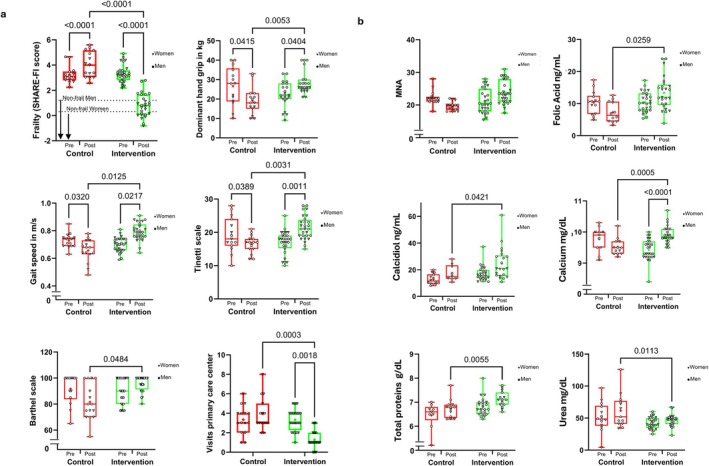
Effects of the multidomain intervention. (a) Functional parameters of participants before and after the intervention including frailty (SHARE‐FI scale), handgrip strength, gait speed, balance (Tinetti scale), Barthel Index, and the number of visits to primary care centers (*n*_cont_ = 14–15, *n*_int_ = 27–28). (b) Nutritional parameters, including MNA score and biochemical data: Blood levels of folic acid, calcidiol, calcium, total protein, and urea (*n*_cont_ = 9–15, *n*_int_ = 22–28). Inverted triangles represent female participants, while circles represent male participants. A two‐sided ANOVA test was used to assess statistically significant differences.

Figure 2a shows that patients who took the supplement and participated in the exercise program exhibited a reduction of −2.5 [−2.9, −2.0] points in frailty according to the SHARE‐FI (Romero‐Ortuno et al. 2011) and also exhibited a reduction in Fried's frailty criteria of −1.8 [−2.2, −1.5] points (Fried et al. 2001) (Table S2). This was accompanied by a remarkable increase in grip strength, gait speed, and balance (as measured by the Tinetti score) following the multidomain intervention. In contrast, the control group deteriorated in all these parameters after 6 months. We also found significant improvement in performance in activities of daily living (Barthel Index). The number of visits to the primary care center was significantly reduced in the intervention group, indicating an optimization of healthcare resource utilization (Figure 2a).

No differences were found at the beginning of the study in the MNA (Figure 2b). An increase in MNA scores toward the well‐nourished category was observed in patients who received the nutritional supplement, although this change did not reach statistical significance. We accompanied the MNA with a blood analysis that included different malnutrition‐related parameters. Figure 2b shows a significant increase in the intervention group's blood calcidiol, total proteins, calcium, and folic acid levels. We also found a decrease in the urea blood levels at the end of the 6 months in the intervention group (Table S3).

No significant changes were observed following the intervention in participants' emotional well‐being (Yesavage scale), social support (Duke scale), cognitive function (MMSE), or perceived quality of life (EQ‐5D). Similarly, the intervention did not significantly improve other geriatric syndromes, including the number of falls, performance in instrumental activities of daily living (Lawton and Brody scale), or frequency of emergency service visits (Table S2).

**FIGURE 3:**
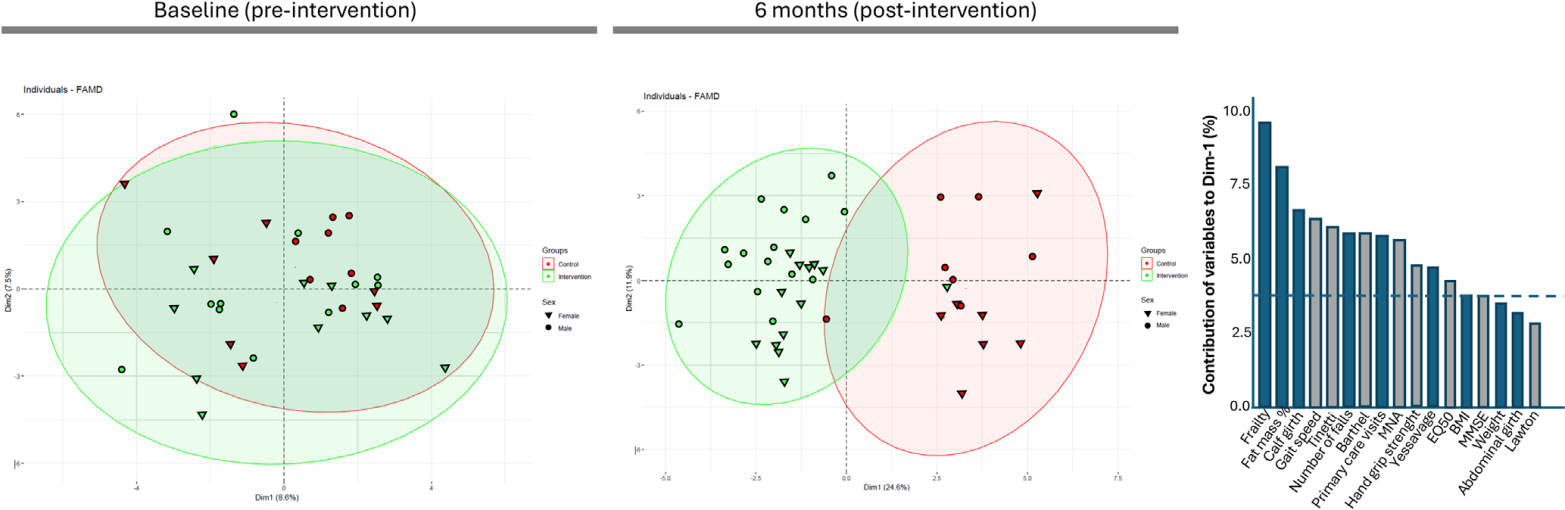
Factor Analysis of Mixed Data, including anthropometric, functional, cognitive, and socio‐emotional variables before (left) and after (right) the intervention. Inverted triangles represent female participants, while circles represent male participants. The histogram displays the variables contributing to Dimension 1 of the FAMD at the final time point of the study, with a contribution > 2.5%. Blue bars represent variables with higher values on the right‐hand side of the diagram (where the control group participants are placed). Gray bars represent variables with higher values on the left hand‐side of the diagram (where the intervention group participants are placed).

Figure 3 presents a FAMD which combines both quantitative and qualitative variables to explore overall patterns and relationships among participants. This multivariate approach allows visualization of how individuals cluster based on their anthropometric, functional, cognitive, and socio‐emotional profiles. On the left‐hand side, the panel displays the distribution of individuals before the intervention (baseline), showing their similarity based on the combined variables. In the central panel, the post‐intervention distribution shows a clear separation along Dimension 1, distinguishing two groups: intervention and control. The right‐hand panel highlights the quantitative variables contributing most strongly to the dimensions with frailty, fat mass percentage, calf girth, gait speed, and the Tinetti scale being the five most influential (Details can be seen in Figure S1).

### 3.3 Comparison of Epigenetic Aging Trajectories Between Intervention and Control Groups

Our study faced a significant challenge during the recruitment phase due to the outbreak of COVID‐19, which significantly impacted sample collection and participant follow‐up (see Limitations of the Study). As a result, we could only obtain complete blood DNA methylation data for 48 samples from 24 participants, each with pre‐ and post‐intervention samples. These included 8 participants in the control group and 16 in the intervention group.

**FIGURE 4:**
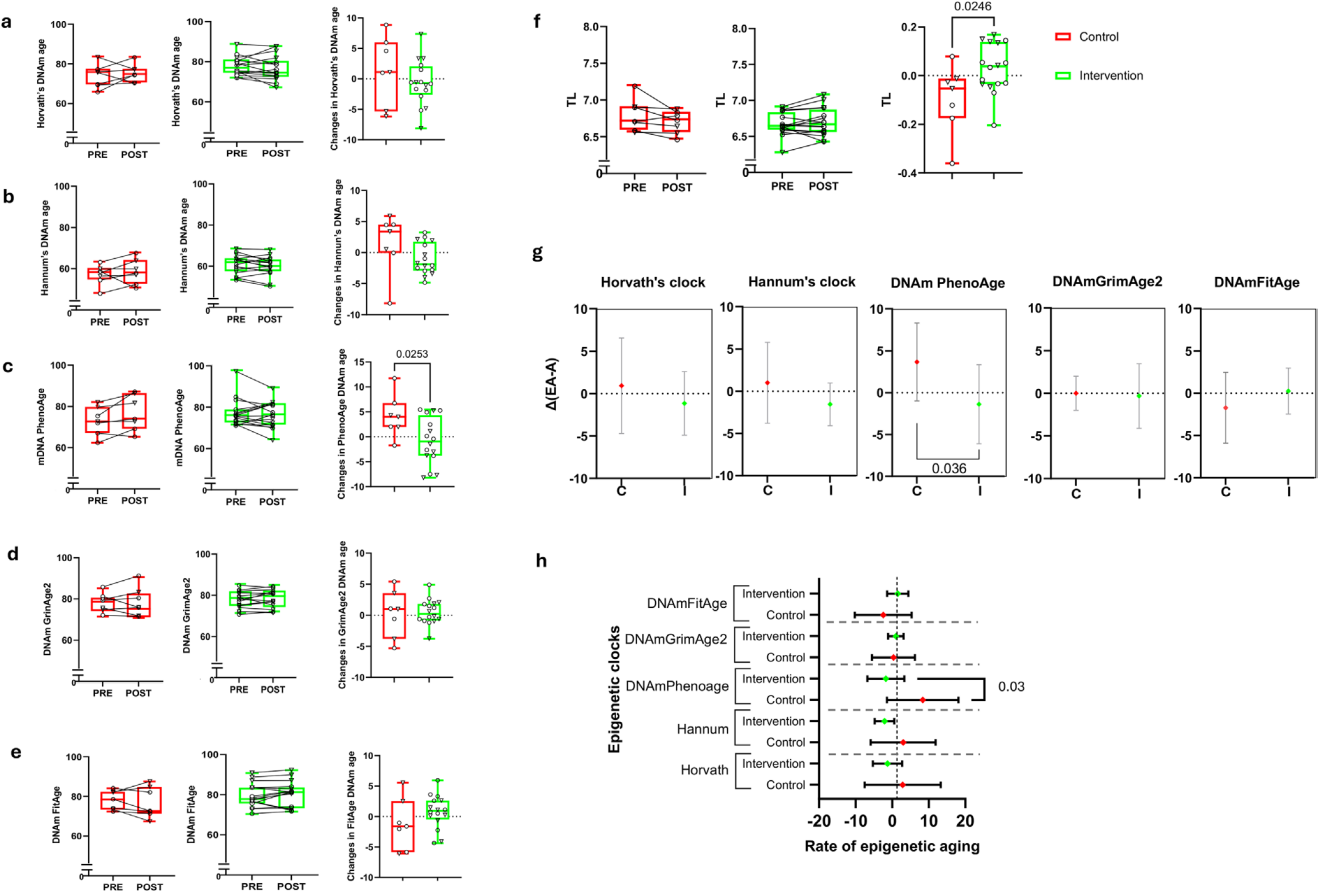
Effects of the intervention on epigenetic age. (a) Effect of the intervention on epigenetic age calculated using Horvath's clock; (b) Hannum's clock; (c) DNAm PhenoAge clock; (d) DNAm GrimAge2 clock; (e) DNAm FitAge clock; (f) DNA methylation‐based telomere length estimation before and after the intervention in the control group (left), in the intervention group (center), and the increase in epigenetic age in both groups (right). For the five epigenetic clocks and estimated telomere length, pre‐ and post‐intervention comparisons were performed using a paired *t*‐test, while between‐group differences in the box plots were analyzed with an independent samples *t*‐test. (g) Variations in the differences between chronological age and epigenetic age, calculated using the five epigenetic clocks. (h) Rate of epigenetic age, defined as the variation in epigenetic age relative to the variation in chronological age. For panels (g and h), mean values and 95% confidence intervals (CI) are represented for each group and each epigenetic clock. An unpaired t‐test assessed statistically significant differences between experimental groups for each clock (*n*_cont_ = 6–7, *n*_int_ = 16).

We calculated five different epigenetic clocks using published algorithms. Figure 4a–e includes box plots (left and center panels) illustrating changes in DNAm clock estimates. The right panels display plots showing the mean change from baseline to the 6‐month follow‐up for the experimental groups. Figure 4a,b show no significant differences in chronological age prediction using Horvath's and Hannum's epigenetic clocks before and after the intervention. Participants in the control group experienced an increase of +4.1 years in biological age, as measured by DNAm PhenoAge, over the 6 months. In contrast, those in the multidomain intervention group did not increase; instead, they exhibited a slight decrease of −0.9 years. This difference was statistically significant (*p* = 0.03) (Figure 4c). Minor changes were found with the DNAm GrimAge version 2 (Lu et al. 2022) and the DNAm FitAge (McGreevy et al. 2023) between the experimental groups, as shown in Figure 4d,e.

We also applied a DNA methylation‐based estimator of telomere length (TL) (Lu et al. 2019). As shown in Figure 4f, TL was reduced in the control group but preserved in the group that underwent the multidomain intervention (*p* = 0.03).

In general terms, the participant's epigenetic ages (EAs) were lower or very similar to their chronological ages (As) at baseline [(EA‐A)_0_ ≤ 0, Table S4] in the control and intervention groups for the five clocks analyzed. Epigenetic age showed a higher decrease after the six‐month follow‐up in the intervention [(EA‐A)_6_ < 0] when compared to the control group in four of the five epigenetic clocks. The only exception to these decreases was the DNAm FitAge (Table S4).

We also assessed the change in the difference between epigenetic and chronological age over the six months calculated as [(EA‐A)_6_–(EA‐A)_0_] (Fahy et al. 2019). For the DNAm PhenoAge clock, the difference between the control and intervention groups reached statistical significance (*p* = 0.04). Only minor changes in this parameter were observed when using the DNAm GrimAge2 and DNAm FitAge clocks. A graphical representation of these differences is provided in Figure 4g.

We correlated the changes in the epigenetic age after six months [(EA‐A)_6_–(EA‐A)_0_] with the chronological age [A_0_] in all the participants. We only found a positive correlation between the two variables in the control group for the DNAm PhenoAge (*r* = 0.82, *p* = 0.04), meaning that the older individuals were more prone to increase their epigenetic age in the control group. Very interestingly, no significant correlations were found among the intervention group (Table S4).

We used the REA to assess the longitudinal dynamics of epigenetic aging in our study (Sehl et al. 2021; Schoepf et al. 2023). REA = 1 means that epigenetic age increases by 1 year per chronological year. REA > 1 indicates that epigenetic aging is advancing faster than chronological aging, and REA < 1 suggests that epigenetic aging is progressing more slowly than chronological aging (Schoepf et al. 2023). Mean REA was > 1 for the first‐generation epigenetic clocks in the control group, while it was < 1 in the intervention group (Figure 4h). The differences reached statistical significance with the DNAm PhenoAge (*p* = 0.03). Although the results showed high variability in the control group, according to this biological clock, the control group's mean REA after the six months was +8.4, whereas the intervention group showed a reduction in this parameter, −1.7 (*p* = 0.03). DNAm GrimAge2 and DNAm FitAge exhibited opposing trends; however, these differences did not reach statistical significance.

We calculated the Epigenetic Age Advancement from the five epigenetic clocks (Figure S2a). Consistent with the results shown in Figure 4a–e, only DNAm PhenoAge demonstrated a statistically significant difference in Epigenetic Age Advancement between the intervention and control groups.

We also quantified the Epigenetic Age Acceleration using the data from the DNAm PhenoAge. Age Acceleration is defined as the difference between DNAm age value and the value predicted by the linear regression model that includes all the participants in the study (control and intervention groups) (Figure S2b, left panel). A positive value of the Age Acceleration indicates that DNA methylation age is higher than that predicted from the regression model. Figure S2b, right panel, shows this is the case in the control group, while the intervention group does not show an increase in Age Acceleration (*p* = 0.55).

We aimed to assess whether there is a correlation between changes in epigenetic age and some of the main variables analyzed. In the control group, the increase in epigenetic age correlated with the increase in waist circumference (*r* = 0.84) (Figure S3). However, no correlation was observed between changes in epigenetic age and frailty as measured by SHARE‐FI or Fried's criteria in either the control or intervention group (see Figures S3 and S4).

No changes in blood cell composition were found in the groups either in basal conditions or after the intervention (see Table S3).

## 4 Discussion

The FINGER trial was the first to apply a multidomain lifestyle intervention, including nutritional guidance and exercise, to target major risk factors for dementia (Ngandu et al. 2015; Rosenberg et al. 2018). Following this work, the European SPRINTT trial demonstrated that a program integrating physical activity, nutritional counseling, and behavioral support can reduce mobility disability in older adults (Bernabei et al. 2022). These successful interventions highlight the potential of multidomain approaches in aging populations and motivated us to explore their application in frail older adults at risk not for cognitive decline, but for functional decline, domains where effective preventive strategies remain limited.

Within the framework of health span research, the benefits of interventions can be quantified using the metrics of aging, which span molecular (biological), phenotypic, and functional domains (Ferrucci et al. 2018).

Our prospective study investigating a multidomain lifestyle intervention versus habitual care in frail older individuals, with pre‐ and post‐intervention assessments, demonstrates significant improvements across all three domains. Our results can be summarized in two major findings:

First, our intervention was associated with a slowing trend in epigenetic aging, as assessed using DNAm PhenoAge. This pattern was observed across the analytical approaches that included changes in methylation age during the intervention, the difference between epigenetic and chronological age (Δ[EA–A]), and the rate of epigenetic aging. Our results are consistent with prior observational studies reporting the sensitivity of second‐generation clocks to lifestyle interventions (Quach et al. 2017; Jain et al. 2022; Fox et al. 2023).

We also found that the age‐associated shortening of telomere length was significantly attenuated in participants receiving the lifestyle intervention. Telomere shortening and alteration in DNA methylation are two well‐established hallmarks of aging related to genomic instability (Zhang et al. 2020). It has been suggested that the modulation of these two processes may be central to the effectiveness of age‐delaying interventions (Zhang et al. 2020; Martínez‐Ezquerro et al. 2024). While our findings provide longitudinal evidence suggesting that lifestyle strategies could influence aging at the molecular‐level without altering the genomic sequence, further studies are needed to confirm the biological significance and long‐term impact of such interventions.

Second, our intervention led to significant improvements in both phenotypic and functional domains. After 6 months, the FAMD revealed a clear separation into two distinct clusters corresponding to the intervention and control groups. The five variables that contributed most strongly to this separation were frailty status, fat mass, calf girth, gait speed, and the Tinetti scale, which are well‐established indicators of physical function and resilience in older adults. These findings suggest that the intervention resulted in multidimensional enhancements in health‐related function extending beyond molecular‐level changes.

While longitudinal data on aging phenotypes are well documented, there is a lack of prospective studies examining human biological aging mechanisms. Our study addresses this gap by incorporating two well‐established biomarkers of biological aging, epigenetic clocks and telomere length, integrated with comprehensive assessments of phenotypic and functional domains in a longitudinal design.

Studies investigating dietary influences on epigenetic aging have primarily focused on caloric restriction. While caloric restriction has demonstrated promising effects in preclinical models and human studies involving healthy adults (Fitzgerald et al. 2021; Rajado et al. 2023; Waziry et al. 2023), its translational applicability to frail older populations remains limited, particularly considering that approximately 23% of European adults over 65 are at risk of malnutrition (Leij‐Halfwerk et al. 2019).

Our approach aimed to improve poor nutritional status and low protein intake, which have been identified as pathophysiologic factors leading to frailty. 4.7% of our sample had malnutrition using the MNA, 72.1% were at risk of malnutrition, and 23.2% were well‐nourished. The nutritional intervention resulted in improvements not only in MNA scores but also in blood biomarkers associated with nutritional status.

Exercise is key to maintaining health and functional independence in older adults. Most exercise interventions targeting older adults rely on non‐tailored and self‐administered protocols primarily because of their scalability and cost‐effectiveness. For instance, in the DO‐HEALTH Bio‐Age trial, it was shown that vitamin D, omega‐3 supplementation, and a simple home‐based exercise program provided additive benefits on biological aging, as measured by DNAm PhenoAge and DNAm GrimAge2, resulting in an age deceleration of 2.9–3.8 months over a 3‐year period (Bischoff‐Ferrari et al. 2025). In 2021, a clinical study involving 43 older adults introduced an eight‐week intervention that integrated caloric restriction, unsupervised physical activity, nutritional supplementation, and lifestyle coaching (Fitzgerald et al. 2021). This intervention reduced epigenetic age by 2 to 3 years, as determined by Horvath's DNA methylation clock. In both trials, the lack of individualization and supervision of the exercise program could limit the effectiveness of such interventions (Liang et al. 2024).

Our intervention outperformed previous studies by incorporating a supervised and individualized exercise program tailored to each participant's fitness level and functional capacity (Tarazona‐Santabalbina et al. 2016; Millan‐Domingo et al. 2022).

In sum, our analysis provides evidence supporting the geroprotective benefits of combining a nutritional supplement with a multicomponent supervised and individualized exercise program in community‐dwelling old, frail individuals.

## 5 Limitations of the Study

Our study faced significant challenges due to COVID‐19 during recruitment. Older, frail participants were reluctant to join a long‐term intervention, and conducting supervised, in‐person exercise was challenging. Consequently, we could not collect biological samples from all enrolled individuals, limiting our molecular results due to a smaller sample size.

As DNA methylation was measured only in whole blood, it remains unclear whether the apparent rejuvenating effect of our intervention can be extrapolated to other tissues. Nonetheless, a previous study demonstrated that epigenetic age acceleration correlates across eleven different tissues within the same individual (Horvath et al. 2022).

No formal multiple comparison adjustments were made for functional or biochemical outcomes to avoid increased type II error with our moderate sample size. Multiple‐testing correction was applied only to methylomic analyses, where it is standard due to the large number of comparisons.

Both exercise and nutrition exert wide‐ranging effects across multiple biological systems. We could not analyze the individual effects of nutritional supplementation and exercise on the outcome variables, as the intervention was intentionally designed as a combined approach. This model needs to be investigated further, particularly concerning the contribution of each component to the overall results. Additionally, the intervention was conducted over a relatively short duration of 6 months, and thus the long‐term sustainability and significance of the observed effects remain uncertain.

## Author Contributions

M.C.G.‐C. and J.V. conceptualized and supervised the study, wrote the manuscript, and granted financial support. G.O.‐G. and F.M.‐D. performed the experiments, analyzed the data, and wrote the manuscript. F.J.T.‐S., E.T.‐T., and J.G. supervised the clinical study and discussed the data. L.G.‐F., C.G.‐D. and E.G.‐T. recruited and evaluated all the patients. M.C. served as a trial coordinator. J.A.C. performed Factor Analysis of Mixed Data. G.C.‐V. and J.L.G.‐G. performed the methylation experiments and analysis. All authors provided final approval of the manuscript

## Funding

This work was supported by the following grants: ISCIII CB16/10/00435 (CIBERFES); PID2022‐142470OB‐I00 and Red EXERNET‐RED DE EJERCICIO FISICO Y SALUD (RED2022‐134800) from the Ministry of Science, Innovation and Universities; PROMETEO (CIPROM/2022/56) from the Consellería de Educación, Universidades, y Empleo de la Generalitat Valenciana; EU Funded H2020‐ DIABFRAIL‐LATAM (Ref: 825546). Generalitat Valenciana has funded part of the equipment employed in this work and co‐financed with FEDER funds (OP FEDER of Comunitat Valenciana 2014–2020). Nestlé Health Science financially supported part of this study.

## Ethics Statement

The study protocol was reviewed and approved by the Research Ethics Committee of the Hospital Universitario de La Ribera, Spain (approval code: HULR22072020). The study was conducted in accordance with the Declaration of Helsinki. All participants provided written informed consent prior to inclusion, and the trial followed a randomized design as approved by the ethics committee.

## Consent

All authors agree with the content of the manuscript and consent to publication.

## Conflicts of Interest

All authors declare independence from the sponsoring body in analyzing results and formulating conclusions. They also report no conflicts of interest with the organizations mentioned above. The authors confirm that AI‐assisted technology, based on OpenAI's GPT‐4 architecture, was used solely to enhance the readability and language of the work in specific paragraphs in the manuscript. This technology was not employed to replace tasks such as generating scientific insights, analyzing and interpreting data, or drawing scientific conclusions. Nestlé Health Science provided financial support for part of this study but had no role in the study design, data collection, data analysis, or data interpretation and did not influence the decision to publish.

## Supporting information


**Figure S1:** FAMD variable factor map. The vectors represent the variables used in the analysis. The direction and length of each vector indicate the contribution of the corresponding variable to the separation of the groups along the factorial axes (right: control, left: intervention).
**Figure S2:** Effects of the intervention on epigenetic age advancement and acceleration. (a) Epigenetic age advancement, calculated as the difference in the mean epigenetic age of each group before and after the intervention. Mean values and 95% confidence intervals (CI) are represented for each group and each epigenetic clock. An unpaired t‐test assessed statistically significant differences between experimental groups for each clock (*n*_cont_ = 6–7, *n*_int_ = 16). (b) (Left) Epigenetic age of participants, estimated using DNAm PhenoAge, from both experimental groups − control (dark red diamonds) and intervention (dark green diamonds) − at baseline. The dashed black line represents the regression line fitted to these data (*y* = 0.6681*x* + 22.39, *R* ^2^ = 0.16), which was used to estimate the epigenetic age of all participants at the end of the study (6 months). Light red (control group) and light green (intervention group) diamonds represent the measured epigenetic age for all subjects at the end of the study. (Right) Epigenetic age acceleration is calculated as the difference between the measured epigenetic age at the end of the study and the estimated epigenetic age based on the baseline regression model for both experimental groups.
**Figure S3:** Correlation plots between changes in epigenetic age (estimated using the DNAm PhenoAge) in the control group and the main variables analyzed in the study. The numbers represent the *R* values for each correlation. Red asterisks indicate statistical significance.
**Figure S4:** Correlation plots between changes in epigenetic age (estimated using the DNAm PhenoAge) in the intervention group and the main variables analyzed in the study. The numbers represent the *R* values for each correlation. Red asterisks indicate statistical significance.

**Table S1:** Baseline characteristics of the participants.
**Table S2:** Additional anthropometric and functional variables.
**Table S3:** Blood analysis of the participants.
**Table S4:** Epigenetic aging characteristics of the study population.

## Data Availability

The data that support the findings of this study are available on request from the corresponding author. The data are not publicly available due to privacy or ethical restrictions.
