# Supplementary material for: A Multidomain Lifestyle Intervention Is Associated With Improved Functional Trajectories and Favorable Changes in Epigenetic Aging Markers in Frail Older Adults: A Randomized Controlled Trial
Source: Aging Cell. 2026 Feb 12;25(2):e70376. doi: 10.1111/acel.70376 (PMC12895478; doi:10.1111/acel.70376)

Supplementary Figure 1

a

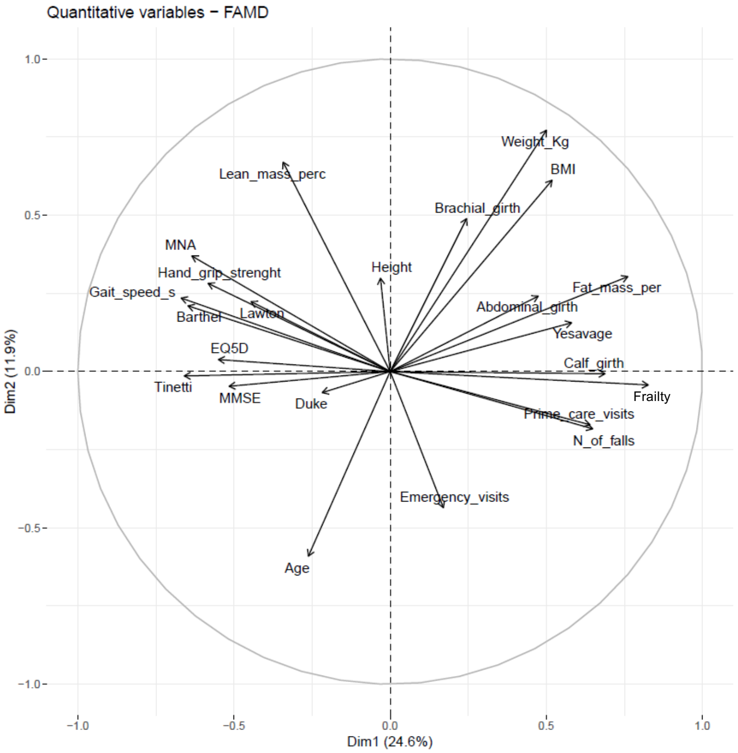

Supplementary Figure 2

a

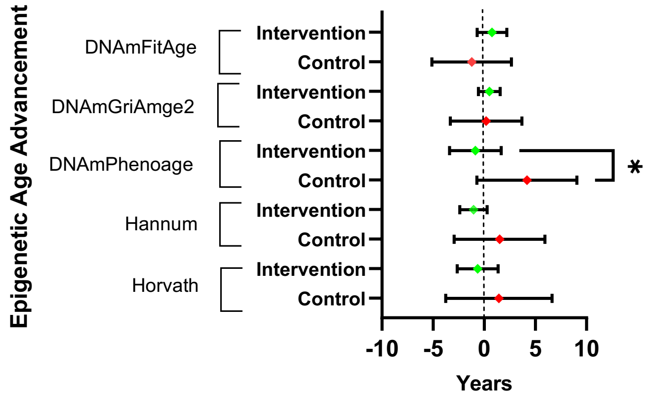

b

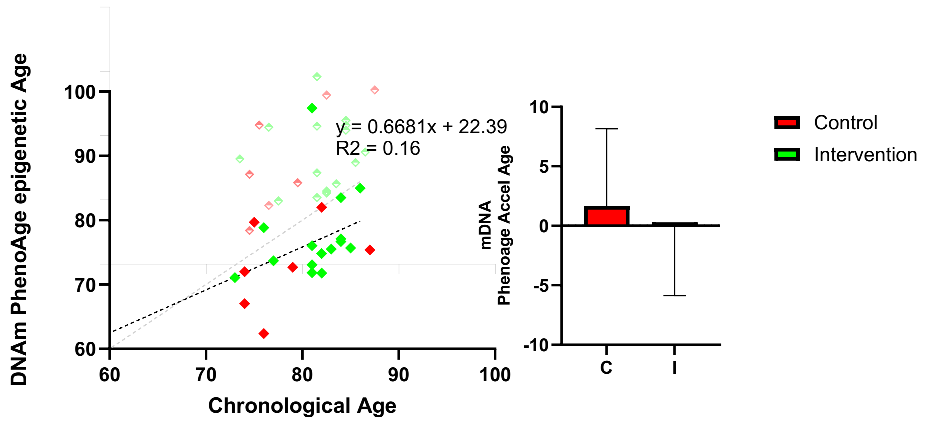

Supplementary Figure 3

CONTROL GROUP

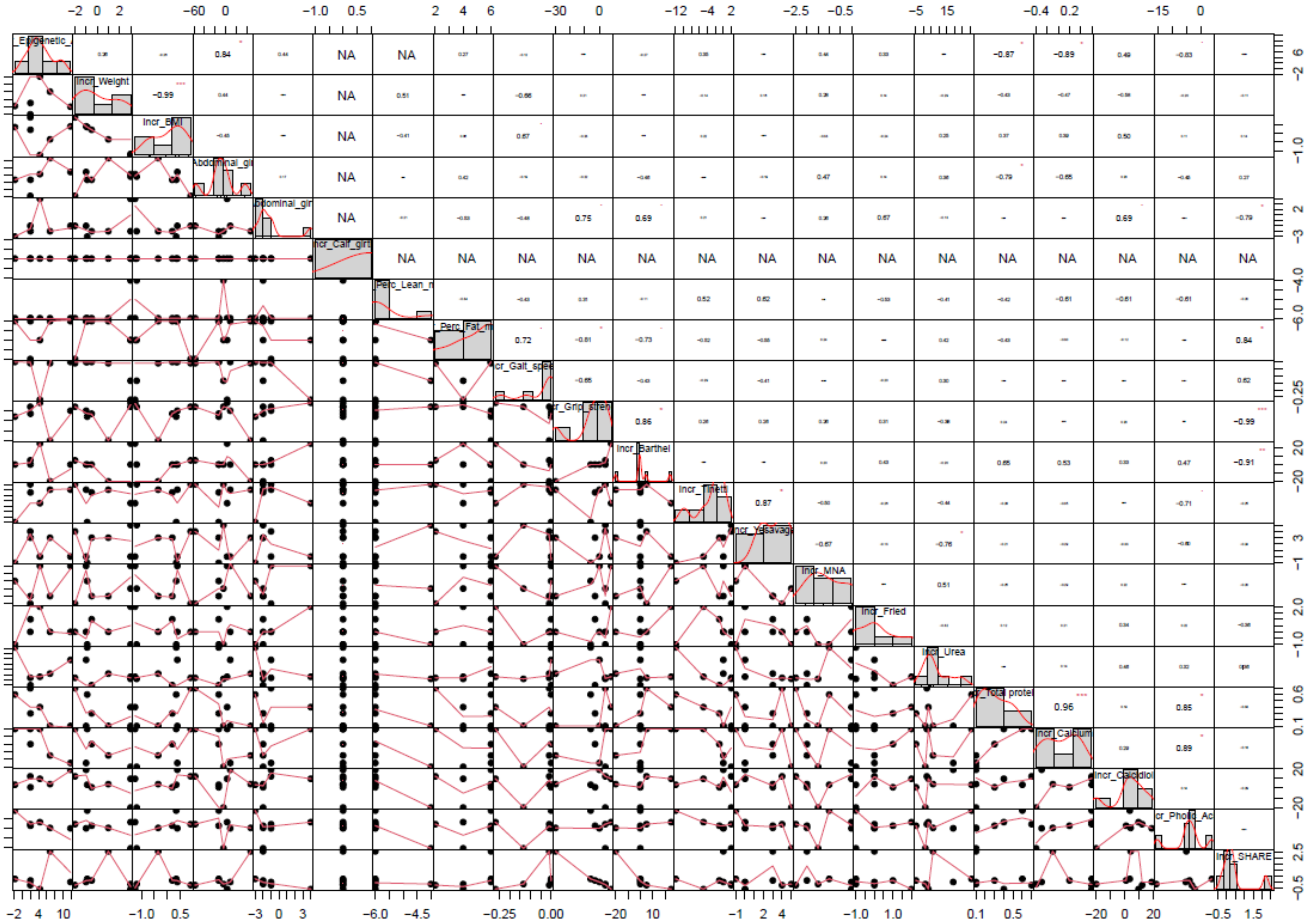

Supplementary Figure 4

INTERVENTION GROUP

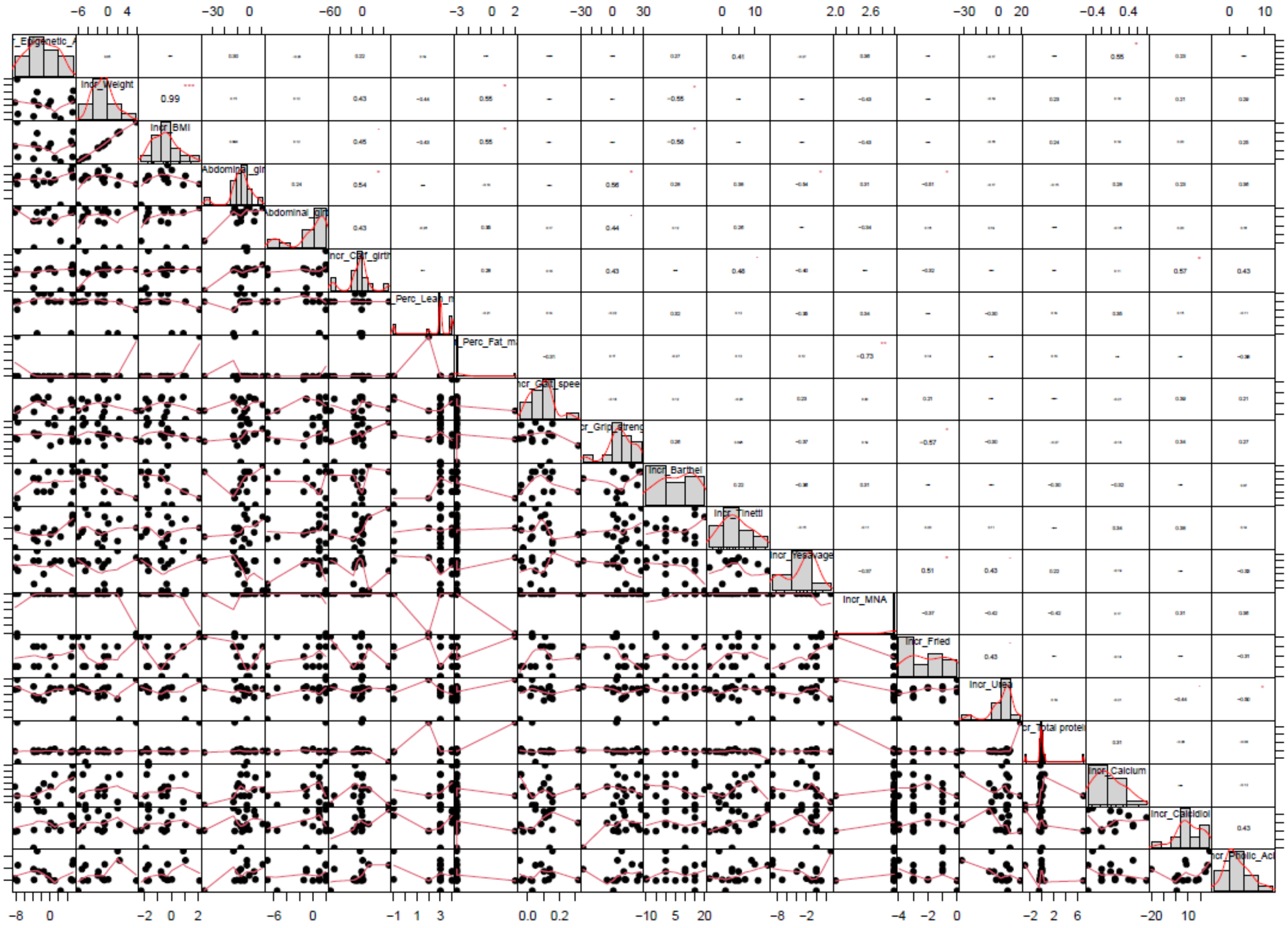

Supplement: Supplementary file 1 — Figure S1: FAMD variable factor map. The vectors represent the variables used in the analysis. The direction and length of each vector indicate the contribution of the corresponding variable to the separation of the groups along the factorial axes (right: control, left: intervention). Figure S2: Effects of the intervention on epigenetic age advancement and acceleration. (a) Epigenetic age advancement, calculated as the difference in the mean epigenetic age of each group before and after the intervention. Mean values and 95% confidence intervals (CI) are represented for each group and each epigenetic clock. An unpaired t‐test assessed statistically significant differences between experimental groups for each clock (n cont = 6–7, n int = 16). (b) (Left) Epigenetic age of participants, estimated using DNAm PhenoAge, from both experimental groups − control (dark red diamonds) and intervention (dark green diamonds) − at baseline. The dashed black line represents the regression line fitted to these data (y = 0.6681x + 22.39, R 2 = 0.16), which was used to estimate the epigenetic age of all participants at the end of the study (6 months). Light red (control group) and light green (intervention group) diamonds represent the measured epigenetic age for all subjects at the end of the study. (Right) Epigenetic age acceleration is calculated as the difference between the measured epigenetic age at the end of the study and the estimated epigenetic age based on the baseline regression model for both experimental groups. Figure S3: Correlation plots between changes in epigenetic age (estimated using the DNAm PhenoAge) in the control group and the main variables analyzed in the study. The numbers represent the R values for each correlation. Red asterisks indicate statistical significance. Figure S4: Correlation plots between changes in epigenetic age (estimated using the DNAm PhenoAge) in the intervention group and the main variables analyzed in the study. The numbers represent the [file ACEL-25-e70376-s002.pdf]
