# Supplementary material for: A Multidomain Lifestyle Intervention Is Associated With Improved Functional Trajectories and Favorable Changes in Epigenetic Aging Markers in Frail Older Adults: A Randomized Controlled Trial
Source: Aging Cell. 2026 Feb 12;25(2):e70376. doi: 10.1111/acel.70376 (PMC12895478; doi:10.1111/acel.70376)
Supplement: Supplementary file 2 — Table S1: Baseline characteristics of the participants. Table S2: Additional anthropometric and functional variables. Table S3: Blood analysis of the participants. Table S4: Epigenetic aging characteristics of the study population. [file ACEL-25-e70376-s001.docx]

**Supplementary Table 1**. Baseline characteristics of the participants

|  |  |  |  | Control  n=19 | Intervention  n=28 | *p* |
| --- | --- | --- | --- | --- | --- | --- |
| Gender n, (%) | |  |  |  |  |  |
|  | Male |  |  | 10 (47.6) | 15 (53.6) | 0.466 |
|  | Female |  |  | 9 (52.4) | 13 (46.4) | 0.365 |
| Age |  |  |  | 80.2 (SD 3.1) | 80.5 (SD 4.3) | 0.781 |
| Marital status n, (%) | |  |  |  |  |  |
|  | Single |  |  | 3 (14.3) | 0 (0) | **<0.0001** |
|  | Married |  |  | 11 (61.9) | 21 (75) | **0.027** |
|  | Widow |  |  | 4 (23.8) | 7 (25) | 0.128 |
| Living with n, (%) | |  |  |  |  |  |
|  | Spouse |  |  | 11 (61) | 21 (75) | **0.034** |
|  | Alone |  |  | 4 (19) | 3 (10.7) | 0.159 |
|  | Children |  |  | 3(19) | 4 (13.3) | 0.487 |
| Conditions n, (%) | |  |  |  |  |  |
|  | Hypertension | |  | 13 (61) | 20 (71.4) | 0.254 |
|  | Hyperlipidemia | |  | 4 (19) | 6 (21.4) | 0.399 |
|  | Diabetes mellitus | |  | 0 (0) | 0 (0) | 0.999 |
|  | Chronic obstructive pulmonary disease | | | 1 (4.8) | 2 (7.1) | 0.523 |
| Fall syndrome n, (%) | | |  | 8 (38.1) | 12 (42.9) | 0.634 |
| No. of falls 6 mo before the study | | | | 0.5 (SD 1.2) | 0.82 (SD 0.6) | 0.138 |
| No. of risk factors for falls | | |  | 5.9 (SD 1.6) | 5.2 (SD 1.8) | 0.884 |
| Hearing impairment | | |  | 0 (0) | 0 (0) | 0.999 |
| Parkinson disease | | |  | 0 (0) | 0 (0) | 0.999 |
| Previous stroke | | |  | 2 (9.5) | 1 (3.6) | 0.477 |
| Arthritis | | |  | 6 (28.6) | 6 (21.4) | 0.146 |
| Heart failure | | |  | 0 (0) | 0 (0) | 0.999 |
| Ischaemic heart disease | | |  | 0 (0) | 0 | 0.999 |
| Renal failure | | |  | 0 (0) | 0 (0) | 0.999 |
| Anxiety depressive disorder | | |  | 14 (66.7) | 16 (57.1) | 0.258 |
| Cancer previous | | |  | 0 (0) | 1 (3.6) | **<0.0001** |
| SHARE-FI | | | | 3.0 (0.7) | 3.2 (0.7) | 0.865 |
| Fried’s Frailty Criteria | | |  | 3.6 (1.1) | 3.2 (0.6) | 0.865 |

|  |  | | |  |  |  | | | | |  |  |
| --- | --- | --- | --- | --- | --- | --- | --- | --- | --- | --- | --- | --- |
| **Supplementary Table 2.**  Additional anthropometric and functional variables. | | | | | | | | | | |  |  |
|  | Baseline (Pre-intervention) | | | | | 6 months (Post-intervention) | | | | | | |
|  | Control | | Intervention | |  | Control | | | Intervention | | |  |
|  | n=14-15 | | n=28 | | *p* | n=14-15 | | | n=27-28 | | | *p* |
|  | Mean | SD | Mean | SD |  | Mean | SD | | Mean | SD | |  |
| Brachial girth (cm) | 30.29 | 4.29 | 31.01 | 5.07 | 0.789 | 30.00 | 2.80 | 29.96 | | 2.78 | | 0.789 |
| Calf girth (cm) | 51.29 | 8.72 | 45.81 | 7.68 | 0.498 | 50.00 | 9.77 | 42.70 | | 5.34 | | 0.498 |
| EQ-5D (points) | 76.33 | 16.09 | 71.79 | 16.57 | 0.378 | 70.33 | 10.77 | 82.68 | | 10.76 | | 0.378 |
| MMSE (points) | 28.47 | 4.02 | 26.07 | 5.68 | 0.942 | 24.07 | 3.20 | 27.50 | | 4.88 | | 0.942 |
| Emergency visits | 0.67 | 0.82 | 0.54 | 0.79 | 0.874 | 0.80 | 1.26 | 0.25 | | 0.65 | | 0.874 |
| Number of falls | 0.47 | 0.74 | 0.71 | 0.71 | 0.998 | 1.20 | 0.56 | 0.29 | | 0.46 | | 0.998 |
| Yesavage scale (points) | 6.47 | 2.75 | 7.00 | 2.88 | 0.724 | 8.47 | 1.88 | 4.89 | | 3.24 | | 0.724 |
| Duke scale (points) | 44.27 | 6.18 | 41.54 | 8.18 | >0.999 | 40.47 | 4.94 | 43.46 | | 7.58 | | >0.999 |
| Lawton & Brody scale (points) | 7.00 | 1.07 | 7.07 | 1.02 | 0.804 | 6.73 | 1.53 | 7.54 | | 0.79 | | 0.804 |
| Frailty (Fried criteria) | 3.47 | 0.52 | 3.14 | 0.85 | 0.987 | 3.53 | 0.83 | 1.25 | | 0.75 | | **0.002** |

|  |  | | |  |  |  | | | | | | |  |  |
| --- | --- | --- | --- | --- | --- | --- | --- | --- | --- | --- | --- | --- | --- | --- |
| **Supplementary Table 3.** Blood biochemical analysis of the participants | | | | | | | | | | | | |  |  |
|  | Baseline (Pre-intervention) | | | | | 6 months (Post-intervention) | | | | | | | | |
|  | Control | | Intervention | |  | Control | | | | Intervention | | | |  |
|  | n=9-15 | | n=28 | | *p* | n=9-15 | | | | n=27-28 | | | | *p* |
|  | Mean | SD | Mean | SD |  | Mean | SD | | | Mean | | SD | |  |
| GFR - mL/min/1.73 m² | 60.4 | 20.5 | 62.9 | 12.7 | 0.286 | 52.5 | 27.7 | | 65.8 | | | 15.9 | | 0.052 |
| Glucose - mg/dL | 112.3 | 25.2 | 111.3 | 28.0 | 0.680 | 114.5 | 19.8 | | 119.5 | | | 31.2 | | 0.783 |
| HbA1c - % | 5.9 | 1.9 | 6.0 | 1.2 | 0.198 | 4.3 | 1.9 | | 5.8 | | | 0.7 | | 0.078 |
| Creatinine - mg/dL | 1.1 | 0.3 | 1.0 | 0.2 | 0.682 | 1.0 | 0.3 | | 1.0 | | | 0.3 | | 0.999 |
| Urea - mg/dL | 50.4 | 19.3 | 42.6 | 11.7 | 0.216 | 57.1 | 26.4 | | 47.5 | | | 10.2 | | **0.011** |
| Total Cholesterol - mg/dL | 155.9 | 36.5 | 177.6 | 41.3 | 0.179 | 147.1 | 41.2 | | 170.8 | | | 39.9 | | 0.057 |
| Triglycerides - mg/dL | 149.1 | 57.6 | 190.3 | 15.5 | 0.269 | 145.2 | 52.9 | | 189.2 | | | 136.1 | | 0.164 |
| Uric Acid - mg/dL | 4.3 | 0.8 | 5.5 | 2.1 | 0.343 | 6.1 | 10.1 | | 5.5 | | | 1.3 | | 0.628 |
| AST - U/L | 21.8 | 9.7 | 26.1 | 9.4 | 0.274 | 19.0 | 9.9 | | 23.1 | | | 13.2 | | 0.682 |
| ALT - U/L | 16.5 | 5.5 | 26.1 | 1.6 | 0.508 | 18.3 | 5.7 | | 29.1 | | | 1.4 | | 0.057 |
| ALP - U/L | 76.6 | 33.9 | 76.6 | 17.4 | 0.695 | 55.8 | 23.7 | | 76.7 | | | 24.0 | | 0.052 |
| GGT - U/L | 26.7 | 32.8 | 28.1 | 24.2 | 0.592 | 19.9 | 18.9 | | 27.4 | | | 24.4 | | 0.053 |
| Total Bilirubin - mg/dL | 0.5 | 0.2 | 0.7 | 0.2 | 0.371 | 0.5 | 0.4 | | 0.6 | | | 0.3 | | 0.783 |
| LDH - U/L | 170.3 | 23.8 | 183.4 | 18.2 | 0.812 | 126.7 | 21.0 | | 170.5 | | | 45.6 | | 0.234 |
| Proteins - g/dL | 6.3 | 0.4 | 5.8 | 0.6 | 0.799 | 5.5 | 0.4 | | 6.5 | | | 0.6 | | **0.006** |
| Albumin - g/dL | 3.8 | 0.3 | 4.3 | 0.3 | 0.345 | 3.5 | 0.4 | | 9.4 | | | 27.1 | | 0.054 |
| Sodium - mmol/L | 141.7 | 2.3 | 135.3 | 0.3 | 0.748 | 130.4 | 4.6 | | 141.0 | | | 3.4 | | 0.682 |
| Potassium - mmol/L | 4.2 | 0.3 | 4.1 | 2.6 | 0.763 | 3.6 | 0.5 | | 4.2 | | | 0.3 | | 0.374 |
| Inorganic Phosphate - mg/dL | 3.0 | 0.6 | 3.2 | 0.3 | 0.861 | 2.5 | 0.6 | | 3.4 | | | 0.4 | | 0.068 |
| Calcium - mg/dL | 9.9 | 2.3 | 9.5 | 0.2 | 0.870 | 7.7 | 0.4 | | 9.8 | | | 0.4 | | **0.005** |
| Chloride - mmol/L | 108.2 | 2.6 | 101.8 | 2.7 | 0.219 | 98.4 | 5.3 | | 106.9 | | | 2.6 | | 0.135 |
| TSH - µIU/mL | 1.5 | 0.7 | 1.9 | 0.4 | 0.251 | 1.7 | 1.6 | | 1.6 | | | 1.0 | | 0.782 |
| Calcidiol - ng/mL | 12.5 | 4.0 | 16.8 | 0.6 | 0.671 | 11.3 | 6.0 | | 22.8 | | | 12.0 | | **0.042** |
| Red Blood Cells - million cells/µL | 4.6 | 0.6 | 4.5 | 0.3 | 0.486 | 4.8 | 0.6 | | 4.5 | | | 0.5 | | 0.238 |
| Hemoglobin - g/dL | 13.7 | 1.3 | 13.3 | 3.6 | 0.781 | 14.0 | 1.5 | | 13.5 | | | 1.2 | | 0.283 |
| Hematocrit - % | 41.9 | 5.2 | 40.7 | 1.4 | 0.725 | 44.3 | 5.0 | | 40.4 | | | 3.5 | | 0.934 |
| MCV - fL | 91.6 | 5.2 | 90.7 | 5.7 | 0.444 | 92.2 | 5.7 | | 86.3 | | | 4.9 | | 0.820 |
| MCH - pg | 34.3 | 17.3 | 29.5 | 0.5 | 0.435 | 29.2 | 2.6 | | 28.8 | | | 1.7 | | 0.065 |
| MCHC - g/dL | 32.4 | 2.3 | 31.4 | 2.3 | 0.755 | 31.6 | 1.2 | | 32.1 | | | 1.6 | | 0.271 |
| RDW - % | 16.2 | 4.6 | 14.0 | 8.2 | 0.536 | 14.8 | 1.6 | | 13.8 | | | 1.2 | | 0.360 |
| White Blood Cells - cells/µL | 7.4 | 1.5 | 6.8 | 8.5 | 0.662 | 7.9 | 2.5 | | 6.9 | | | 2.0 | | 0.307 |
| Neutrophils - % | 63.2 | 8.0 | 58.5 | 2.5 | 0.391 | 64.4 | 13.1 | | 58.9 | | | 6.6 | | 0.380 |
| Lymphocytes - % | 25.1 | 6.9 | 26.4 | 1.2 | 0.364 | 24.7 | 10.9 | | 26.1 | | | 6.3 | | 0.645 |
| Monocytes - % | 6.1 | 1.1 | 6.0 | 1.1 | 0.207 | 5.9 | 1.0 | | 5.9 | | | 1.6 | | 0.387 |
| Eosinophils - % | 3.1 | 1.9 | 2.4 | 1.9 | 0.280 | 2.7 | 2.0 | | 2.7 | | | 1.7 | | 0.346 |
| Basophils - % | 0.6 | 0.2 | 0.6 | 0.7 | 0.865 | 0.5 | 0.3 | | 0.7 | | | 0.5 | | 0.110 |
| % Neutrophils - % | 4.4 | 1.4 | 4.3 | 7.0 | 0.678 | 5.3 | 2.6 | | 4.1 | | | 1.5 | | 0.065 |
| % Lymphocytes - % | 1.8 | 0.4 | 2.0 | 1.6 | 0.390 | 1.7 | 0.6 | | 2.0 | | | 0.8 | | 0.993 |
| % Monocytes - % | 0.5 | 0.1 | 0.4 | 1.4 | 0.735 | 0.5 | 0.2 | | 0.5 | | | 0.3 | | 0.658 |
| % Eosinophils - % | 0.2 | 0.1 | 0.2 | 0.3 | 0.195 | 0.3 | 0.4 | | 0.2 | | | 0.1 | | 0.944 |
| % Basophils - % | 0.0 | 0.0 | 0.0 | 1.4 | 0.280 | 0.0 | 0.0 | | 0.0 | | | 0.1 | | 0.490 |
| Platelets - thousand/µL | 200.8 | 45.8 | 214.4 | 8.0 | 0.295 | 217.4 | 37.8 | | 173.6 | | | 36.9 | | 0.495 |
| MPV - fL | 8.9 | 0.9 | 8.0 | 0.1 | 0.847 | 9.3 | | 0.9 | 9.0 | | 0.9 | | | 0.364 |
| Iron - µg/dL | 69.6 | 26.7 | 76.0 | 21.3 | 0.666 | 57.9 | | 45.9 | 55.3 | | 23.6 | | | 0.389 |
| Transferrin Saturation - % | 310.8 | 119.6 | 355.6 | 116.4 | 0.869 | 182.9 | | 91.2 | 325.3 | | 53.7 | | | 0.794 |
| Iron Binding Capacity - µg/dL | 18.6 | 8.0 | 23.2 | 7.4 | 0.635 | 8.1 | | 11.1 | 15.5 | | 7.2 | | | 0.460 |
| Ferritin - ng/mL | 67.3 | 75.0 | 82.6 | 67.4 | 0.845 | 76.0 | | 104.1 | 62.2 | | 53.7 | | | 0.249 |
| Vitamin B12 - pg/mL | 364.8 | 186.2 | 471.3 | 36.3 | 0.582 | 346.1 | | 166.5 | 464.2 | | 342.0 | | | 0.164 |
| Folic Acid - ng/mL | 10.1 | 3.7 | 10.1 | 5.5 | 0.782 | 6.0 | | 3.6 | 12.8 | | 5.2 | | | **0.026** |
| Transferrin - mg/dL | 242.5 | 51.1 | 253.1 | 17.4 | 0.486 | 129.7 | | 64.8 | 217.0 | | 31.4 | | | 0.052 |

**Supplementary Table 4.** Epigenetic aging characteristics of the study population.

|  | | **Control** | | **Multidomain Intervention** | |
| --- | --- | --- | --- | --- | --- |
| 1^st^ Generation Epigenetic Clocks | **DNAm Horvath** | (EA-A)_0_ | -4.27 ± 6.00 | (EA-A)_0_ | -3.03 ± 5.41 |
|  |  | (EA-A)_6_ | -3.33 ± 3.98 | (EA-A)_6_ | -4.17 ± 6.24 |
|  |  | (EA-A)_6_ - (EA-A)_0_ | 0.93 ± 5.63 | (EA-A)_6_ - (EA-A)_0_ | -1.14 ± 3.75 |
|  |  | Correlation (EA-A)_6_ - (EA-A)_0_ and A_0_ | r = 0.086  p = 0.85 | Correlation (EA-A)_6_ - (EA-A)0 and A0 | r = 0.24  p = 0.38 |
|  |  | Correlation (EA-A)_6_ - (EA-A)_0_ and (EA-A)_0_ | r = -0.77  **p = 0.04** | Correlation (EA-A)_6_ - (EA-A)_0_ and (EA-A)_0_ | r = -0.10  p = 0.69 |
|  | **DNAm Hannum** | (EA-A)_0_ | -21.19 ± 4.83 | (EA-A)_0_ | -19.76 ± 5.01 |
|  |  | (EA-A)_6_ | -20.18 ± 3.45 | (EA-A)_6_ | -21.31 ± 5.66 |
|  |  | (EA-A)6 - (EA-A)0 | 1.00 ± 4.79 | (EA-A)_6_ - (EA-A)_0_ | -1.55 ± 2.53 |
|  |  | Correlation (EA-A)_6_ - (EA-A)_0_ and A_0_ | r = 0.52  p = 0.23 | Correlation (EA-A)_6_ - (EA-A)_0_ and A_0_ | r = -0.23  p = 0.38 |
|  |  | Correlation (EA-A)_6_ - (EA-A)_0_ and (EA-A)_0_ | r =0.56  p = 0.06 | Correlation (EA-A)_6_ - (EA-A)_0_ and (EA-A)_0_ | r = 0.02  p = 0.93 |
| 2^nd^ Generation Epigenetic Clocks | **DNAm PhenoAge** | (EA-A)_0_ | -5.13 ± 6.47 | (EA-A)_0_ | -3.57 ± 6.63 |
|  |  | (EA-A)_6_ | -2.05 ± 5.91 | (EA-A)_6_ | -4.94 ± 6.15 |
|  |  | (EA-A)_6_ - (EA-A)_0_ | **3.08 ± 4.53** | (EA-A)_6_ - (EA-A)_0_ | **-1.37 ± 4.73 *** |
|  |  | Correlation (EA-A)_6_ - (EA-A)_0_ and A_0_ | r = 0.82  **p=0.04** | Correlation (EA-A)_6_ - (EA-A)_0_ and A_0_ | r = 0.03  p = 0.92 |
|  |  | Correlation (EA-A)_6_ - (EA-A)_0_ and (EA-A)_0_ | r = -0.53  p=0.28 | Correlation (EA-A)_6_ - (EA-A)_0_ and (EA-A)_0_ | r = -0.45  p = 0.08 |
|  | **DNAm GrimAge2** | (EA-A)_0_ | 0.26±4.33 | (EA-A)_0_ | -3.02 ±4.10 |
|  |  | (EA-A)_6_ | -0.05±4.72 | (EA-A)_6_ | -3.01 ±4.10 |
|  |  | (EA-A)_6_ - (EA-A)_0_ | -0.31±3.80 | (EA-A)_6_ - (EA-A)_0_ | -0.01 ± 2.00 |
|  |  | Correlation (EA-A)_6_ - (EA-A)_0_ and A_0_ | r = 0.66  p =0.10 | Correlation (EA-A)_6_ - (EA-A)_0_ and A_0_ | r = 0.12  p = 0.66 |
|  |  | Correlation (EA-A)_6_ - (EA-A)_0_ and (EA-A)_0_ | r =0.33  p =0.47 | Correlation (EA-A)_6_ - (EA-A)_0_ and (EA-A)_0_ | r = -0.24  p = 0.36 |
| 3^rd^ Generation Epigenetic Clocks | **DNAm FitAge** | (EA-A)_0_ | 0.03 ± 4.58 | (EA-A)_0_ | -0.94 ± 5.35 |
|  |  | (EA-A)_6_ | -1.69 ± 7.09 | (EA-A)_6_ | -0.69 ± 5.12 |
|  |  | (EA-A)_6_ - (EA-A)_0_ | -1.72 ± 4.19 | (EA-A)_6_ - (EA-A)_0_ | 0.25 ± 2.71 |
|  |  | Correlation (EA-A)_6_ - (EA-A)_0_ and A_0_ | r = 0.19  p = 0.68 | Correlation (EA-A)_6_ - (EA-A)_0_ and A_0_ | r = 0.32  p = 0.23 |
|  |  | Correlation (EA-A)_6_ - (EA-A)_0_ and (EA-A)_0_ | r = 0.39  p = 0.51 | Correlation (EA-A)_6_ - (EA-A)_0_ and (EA-A)_0_ | r = -0.33  p = 0.20 |

EA = epigenetic age; A = chronological age; 0 = at zero months (trial onset); 6 = at 6 months after the onset of the intervention (trial end); 0 = age at trial onset; all results are given in years. *p < 0.05 for the comparison
